# Chromosome-scale genome assemblies of sexually dimorphic male and female *Acrossocheilus fasciatus*

**DOI:** 10.1038/s41597-024-03504-9

**Published:** 2024-06-21

**Authors:** Yixin Yuan, Tianxing Zhong, Yifei Wang, Jinquan Yang, Lang Gui, Yubang Shen, Jiajun Zhou, Yu-Wen Chung-Davidson, Weiming Li, Jinkai Xu, Jiale Li, Mingyou Li, Jianfeng Ren

**Affiliations:** 1 Key Laboratory of Freshwater Aquatic Genetic Resources certificated by the Ministry of Agriculture and Rural Affairs, Shanghai Ocean University, Shanghai, 201306 China; 2 Zhejiang Forest Resource Monitoring Center, Hangzhou, 310020 China; 3 Department of Fisheries and Wildlife, Michigan State University, East Lansing, MI 48824 USA; 4 Huangshan Dingxin Ecological Agriculture Co., Ltd, Huangshan, 245431 China

**Keywords:** Genome, Ichthyology

## Abstract

*Acrossocheilus fasciatus* is a stream-dwelling fish species of the Barbinae subfamily. It is valued for its colorfully striped appearance and delicious meat. This species is also characterized by apparent sexual dimorphism and toxic ovum. Biology and aquaculture researches of *A. fasciatus* are hindered by the lack of a high-quality reference genome. Here, we report chromosome-level genome assemblies of the male and female *A. fasciatus*. The HiFi-only genome assemblies for both female and male individuals were 899.13 Mb (N50 length of 32.58 Mb) and 885.68 Mb (N50 length of 33.06 Mb), respectively. Notably, a substantial proportion of the assembled sequences, accounting for 96.15% and 98.35% for female and male genomes, respectively, were successfully anchored onto 25 chromosomes utilizing Hi-C data. We annotated the female assembly as a reference genome and identified a total of 400.62 Mb (44.56%) repetitive sequences, 27,392 protein-coding genes, and 35,869 ncRNAs. The high-quality male and female reference genomes will provide genomic resources for developing sex-specific molecular markers, inform single-sex breeding, and elucidate genetic mechanisms of sexual dimorphism.

## Background & Summary

**Fig. 1:**
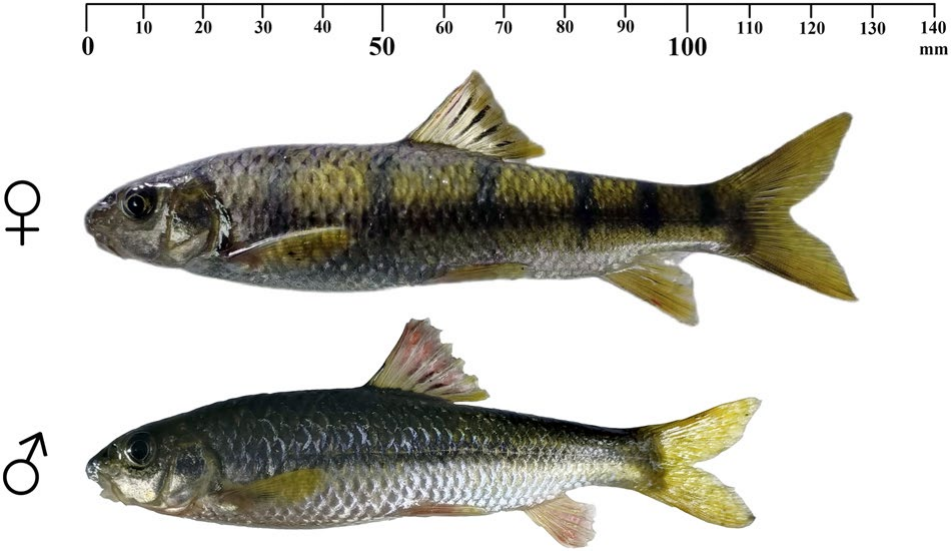
One-and-a-half-year-old *Acrossocheilus fasciatus* with sexual dimorphism.

The Barbinae is a subfamily of the Cyprinidae that is the largest family of freshwater fishes. This subfamily contains the most complex and diverse fish groups within the Cyprinidae^1^. Their morphologies and habits are highly diverse. For example, *Sinocyclocheilus rhinocerous* dwells in caves and has evolved relevant traits^2^. Genome sequences of several Barbinae species, including three species of genus *Sinocyclocheilus* (*S. grahami, S. rhinocerous*, and *S. anshuiensis*), *Poropuntius huangchuchieni*, *Puntigrus tetrazonahas*, and *Onychostoma macrolepis*, have been deciphered, largely due to their phylogeny features and notable evolutionary status^2–4^. Most of the species in the Barbinae had undergone whole genome duplication after the third round of teleost-specific genome duplication (TGD) event that generated tetraploid even hexaploid^5^. However, some species remain diploids that retain the original chromosome number 2n=50, such as *O. macrolepis, P. huangchuchieni and P. tetrazonahas*^3,4,6^. *Acrossocheilus fasciatus* is also a diploid species in the Barbinae, with chromosome number 2n=50^7^. It is mainly found in streams south of the Yangtze River and is extremely popular with recreational fisheries due to its colorful appearance with six dark stripes. It is a local delicacy and is considered highly nutritious^8^ by people in southeast China, especially in Zhejiang Province. However, because of its small size and slow growth rate^9^, this fish is always in short supply and has great market prospects. In addition, *A. fasciatus* is ichthyootoxic, with toxic ova^10^. The structures of the toxins remain unknown. Furthermore, it is sexually dimorphic in both body mass and appearance (Fig. 1). The weight of a two-year-old mature female is approximately 1.5 times that of the mature male^11^. In mature males, the six black transverse stripes gradually faded with the appearance of secondary sex characteristics such as the pearl organs and redness of the abdomen, whereas the females always retain the transverse stripes.

Despite its biological and economic importance, the genomic resources of *A. fasciatus* are limited. Several studies on *A. fasciatus* were focused on the mitochondrial DNA or transcriptomes^12–16^. In this study, we sequenced and annotated the chromosome-scale genome assemblies of the male and female *A. fasciatus* using PacBio HiFi reads and high-throughput chromosome conformation capture (Hi-C) technologies. The genome size of female *A. fasciatus* was estimated to be about 880.6 Mb through k-mer frequency distribution analysis with 126.33 Gb (~143 × ) Illumina clean data. The female and male genomes were independently assembled into contigs with PacBio HiFi reads. The female genome assembly spans 899.13 Mb with a contig N50 length of 32.58 Mb using 62.01 Gb (~70 × ) PacBio HiFi clean reads. The male genome spans 885.68 Mb with a contig N50 length of 33.06 Mb using 97.67 Gb (~111 × ) of HiFi clean reads. 96.15% and 98.35% of contig sequences of the female (contigs N50 length = 32.35 Mb; scaffolds N50 length = 33.86 Mb) and male (contigs N50 length = 32.84 Mb; scaffolds N50 length = 33.78 Mb) genomes were anchored onto 25 chromosomes using Hi-C data (Supplementary Table 1). Finally, the female genome was annotated as a reference genome with 44.56% (400.62 Mb) of repetitive sequences, 27,392 protein-coding genes, and 35,869 ncRNAs. The female and male genome assemblies reported here provide genomic resources for development of sex-specific molecular markers and single-sex breeding as well as a better understanding of the mechanisms of sexual dimorphism.

## Methods

### Sample collection

Two-year-old female and male adults of *A. fasciatus* were randomly sampled from the second-generation progeny of selective breeding performed in Dingxin Ecological Agriculture Co., Ltd. (Xiuning County, Huangshan City of Anhui Province, China). The sampled fish were euthanized with MS-222 (Sigma-Aldrich, #A5040) and dissected on ice. Eight tissues including the brain, gill, heart, intestine, liver, ovary, muscle, and skin of one female (body length = 16.23 cm, body weight = 43.56 g) were collected and immediately frozen in liquid nitrogen and then stored at −80 °C until DNA and RNA extraction. The blood and muscle tissues of one male (body length = 13.05 cm, body weight = 26.73 g) were collected for DNA extraction.

### DNA extraction and sequencing for genomes

The high-molecular weight (HMW) genomic DNA from the female muscle and the male blood of *A. fasciatus* was extracted using the phenol/chloroform method^17^. The quality and quantity of the extracted DNA were assessed using 1.0% agarose gel electrophoresis and a Qubit 4.0 fluorometer (Thermo Fisher Scientific, USA).

For PacBio sequencing, the high-quality DNA (main band > 30 kb) was randomly interrupted into 15–18 kb size fragments by a Covaris g-TUBE (Woburn, Massachusetts, USA), and then the SMRTbell libraries were constructed using the PacBio HiFi Express Template Prep Kit 2.0 according to the manufacturer’s instruction^18^ (Pacific Biosciences, Menlo Park, CA, USA). For the female genome assembly, we generated two cells of HiFi clean reads with 62.01 Gb (~70 × ) data and an N50 read length of 14.12 kb using PacBio Sequel IIe platform. For the male genome assembly, we generated only one cell of HiFi reads with 97.67 Gb (~111 × ) data and an N50 read length of 13.96 kb using PacBio Revio platform (Table 1). For Illumina sequencing, the DNA was randomly interrupted into ~350 bp fragments using the Covaris ultrasonic crusher. Libraries were constructed using NEBNext^®^ Ultra^TM^ DNA Library Prep Kit for Illumina (NEB, #E7370L) and sequenced on the Novaseq 6000 platform (Illumina, Inc., San Diego, CA, USA) with paired-end (PE) 150 bp model. We also obtained 126.33 Gb (~143 × ) of Illumina short reads to survey the female genome (Table 1).

**Table 1:** Statistics of the sequencing data for *A. fasciatus* genomes.

Individual	Tissue type	Data type	Platform	Data size (Gb)
Female	Muscle	WGS long reads	PacBio Sequel IIe	62.01
	Muscle	WGS short reads	Illumina Novaseq 6000	126.33
	Muscle	Hi-C	Illumina Novaseq 6000	137.24
	Brain, gill, heart, intestine, liver, ovary, muscle, skin	RNA short reads	Illumina Novaseq 6000	56.32
	Brain, gill, heart, intestine, liver, ovary, muscle, skin	RNA long reads	PacBio Sequel IIe	20.25
Male	Blood	WGS long reads	PacBio Revio	97.67
	Muscle	Hi-C	Illumina Novaseq 6000	104.69

For genome scaffolding, Hi-C libraries were prepared using muscle tissues from both female and male individuals for PacBio genome sequencing. The Hi-C library construction, including cell crosslinking, cell lysis, chromatin digestion (*Mbo*I), biotin labeling, proximal chromatin DNA ligation and DNA purification, was performed according to the standard protocol described previously^19,20^. After quality control assessment by Agilent 2100 Bioanalyzer and qPCR test, the resulting Hi-C libraries were subjected to sequencing with PE 150 bp model on Illumina Novaseq. 6000 platform. As a result, a total of 137.24 Gb (~152 × ) and 104.69 Gb (~116 × ) raw read data were generated for the female and male genome, respectively (Table 1).

### RNA extraction and transcriptome sequencing

Eight sampled tissues, including the brain, gill, heart, intestine, liver, ovary, muscle, and skin of the female *A. fasciatus* were each extracted for total RNA using TRIzol^TM^ reagent (Thermo Fisher Scientific, USA). The resulting RNAs were treated with DNase I (NEB, USA) to remove the genomic DNA.

To facilitate genome annotation, both Iso-Seq and RNA-Seq were performed. For PacBio Iso-Seq, the RNAs were mixed equimolarly and subjected to sequencing. Specifically, the concentration, integrity, and purity of the RNA isolated from each tissue of the female were confirmed using Qubit, Agilent 2100 and Nanodrop, then pooled together at an equimolar concentration. A double-stranded cDNA library was prepared with SMARTer^®^ PCR cDNA Synthesis Kit (Clontech, USA). Subsequently, the cDNA library was sequenced using the PacBio Sequel IIe platform. After filtering and treating using SMRTlink v11.0 (https://www.pacb.com/support/software-downloads/) with parameters–minLength=50, a total of 20.25 Gb of subreads data were generated (Table 1). For Illumina RNA-seq, eight cDNA libraries from the aforementioned tissues were constructed independently and sequenced using Illumina NovaSeq 6000. A total of 56.32 Gb clean data were generated after removing reads containing adapters, reads with more than 10% unknown nucleotides (Ns) or low-quality bases (more than 20% bases with Phred quality < 5) (Table 1).

### *De novo* genome assembly with PacBio HiFi reads and Hi-C technologies

Before *de novo* assembly, the size of the female genome was estimated with k-mer analysis of Illumina reads. The Illumina clean reads were filtered to remove redundancy with in-house script redup.v2 developed by Novogene (Beijing, China), and utilized to calculate the k-mer frequency with k=17 using Jellyfish v2.2.7^21,22^. Based on the formula: genome size = k-mer number/peak depth, the female genome size of *A. fasciatus* was estimated to be 880.6 Mb, with a heterozygous ratio of 0.53% and repeat rate of 47.82% (Supplemental Fig. 1).

PacBio HiFi reads from the female and the male individuals were assembled into the female contigs and the male contigs using Hifasm v0.16.1^23^ with default parameters. A total of 110 female contigs were built with a total length of 899,126,031 bp and an N50 length of 32.58 Mb. And a total of 174 male contigs were built with a total length of 885,680,593 bp and an N50 length of 33.06 Mb.

**Fig. 2:**
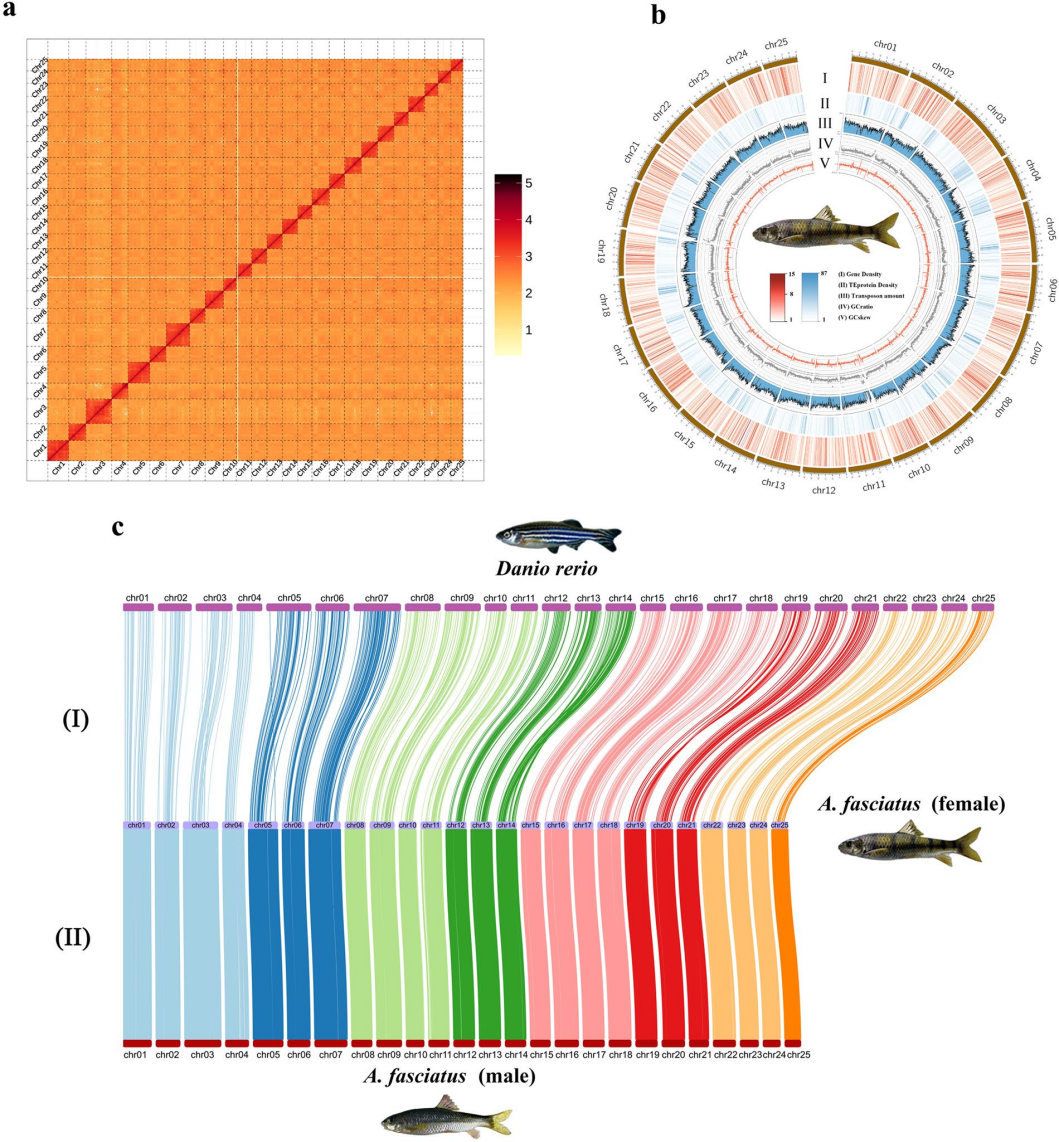
Genome assembly and chromosome synteny analysis. (**a**) Hi-C interactive heatmap of the female genome. (**b**) Genomic landscape of female *A. fasciatus*. The Circos plot illustrates from outside to inside (I) gene density, (II) TE protein density, (III) transposon amount, (IV) GC ratio, and (V) GCskew. (**c**) Chromosome synteny analyses. Chromosome synteny between *Danio rerio* and female *A. fasciatus* (I), and between female and male *A. fasciatus* (II).

The Hi-C raw reads were processed to remove paired reads that contain adapters or low-quality bases (more than 20% bases with Phred quality <5), and quality-controlled by HiCUP^24^. Subsequently, the contigs were anchored into 25 pseudo-chromosomes using ALLHiC pipeline^25^ with the clean Hi-C data (Fig. 2a). Juicebox software was used to correct chromosome interaction strength artificially (Supplemental Fig. 2)^26^. As a result, 84 scaffolds of the female genome were generated with a total length of 899,129,631 bp and an N50 length of 33.86 Mb, of which 96.15% (864,515,734 bp) was anchored onto 25 chromosomes (Tables 2, 3). 167 scaffolds of the male genome were generated with a total length of 885,681,293 bp and an N50 length of 33.78 Mb, of which 98.35% (871,084,321 bp) was anchored onto 25 chromosomes (Tables 2, 3). Finally, we obtained the high-quality chromosome-level male and female reference genomes with Hi-C technologies^20^ for genome characters analysis (Fig. 2b).

**Table 2:** Statistics of the 25 chromosomes in both female and male genomes of *A. fasciatus*.

Female			Male		
Chromosome ID	Contig number	Sequence length	Chromosome ID	Contig number	Sequence length
Chr01	4	43,112,031	Chr01	1	43,405,856
Chr02	2	36,507,323	Chr02	2	36,680,643
Chr03	4	53,107,739	Chr03	1	54,788,890
Chr04	3	33,558,333	Chr04	2	35,510,000
Chr05	3	44,991,246	Chr05	1	45,215,713
Chr06	2	34,145,683	Chr06	1	34,229,423
Chr07	2	48,819,166	Chr07	2	49,012,890
Chr08	2	32,158,869	Chr08	1	31,606,299
Chr09	2	37,614,112	Chr09	1	38,142,591
Chr10	4	27,383,622	Chr10	3	28,706,205
Chr11	3	31,932,368	Chr11	1	31,298,002
Chr12	3	31,684,124	Chr12	1	31,897,254
Chr13	2	32,619,312	Chr13	1	32,795,843
Chr14	2	31,797,777	Chr14	1	32,116,842
Chr15	2	30,090,052	Chr15	1	30,504,684
Chr16	2	36,381,764	Chr16	2	36,003,676
Chr17	2	31,965,306	Chr17	1	32,838,721
Chr18	2	34,292,799	Chr18	1	33,775,926
Chr19	2	33,862,838	Chr19	1	33,059,052
Chr20	2	34,223,592	Chr20	2	34,742,167
Chr21	2	29,890,321	Chr21	1	29,700,910
Chr22	2	34,076,605	Chr22	1	34,521,066
Chr23	3	28,176,661	Chr23	1	29,170,000
Chr24	2	26,755,058	Chr24	1	26,474,184
Chr25	2	25,369,033	Chr25	1	24,887,484
Total	61	864,515,734	Total	33	871,084,321

**Table 3:** Statistics of scaffolds anchored in both the female and male genomes of *A. fasciatus*.

Female			Male		
Type	Scaffold number	Total length	Type	Scaffold number	Total length
Placed	25	864,515,734	Placed	25	871,084,321
Unplaced	59	34,613,897	Unplaced	142	14,596,972
Total	84	899,129,631	Total	167	885,681,293
Anchor ratio	96.15%		Anchor ratio	98.35%	

### Genomic synteny analysis

To assign the chromosome ID of *A. fasciatus* genomes and assess the accuracy of genome assemblies, we performed the genomic synteny analysis between zebrafish *Danio rerio*, and the female and male *A. fasciatus*. For synteny analysis between the assemblies of zebrafish and female *A. fasciatus*, Mummer^27^ (v4.0.0beta2) was used to match the maximal unique sequences between the genomes with parameter “–mincluster 500”. The matched sequence sets were filtered by removing the sets with sequence similarity less than 80%. For synteny analysis between the female and the male assemblies of *A. fasciatus*, the matched sequence sets were filtered by removing the sets with sequence similarity of less than 95% and length less than 10 kb. Genomic synteny graphs were generated with the matched sets using RectChr v1.36 (https://github.com/BGI-shenzhen/RectChr) (Fig. 2c). The synteny graphs indicated a moderate level of collinearity with minor rearrangements between the genomes of zebrafish and *A. fasciatus*, and the genome assemblies of the female and male *A. fasciatus* are remarkably accurate. No obvious chromosome structure variation was observed between female and male genomes through synteny analysis.

### Repeat annotation of the female genome

The repeat sequences mainly consisted of interspersed repeats (mainly transposable elements, TEs) and tandem repeats. The repeat sequences of TEs in the female *A. fasciatus* genome were identified using a strategy combing homology alignment and *ab initio* search. Tandem repeats were predicted *ab initio* using TRF^28^. Firstly, the homolog prediction of TEs was based on Repbase^29^ database employing RepeatMasker and RepeatProteinMask^30^ (https://www.repeatmasker.org/) with default parameters. Secondly, *de novo* repetitive elements were identified by LTR_FINDER^31^, RepeatScout^32^, and RepeatModeler^33^ with the default parameters. All repeat sequences with length > 100 bp and a gap ‘N’ less than 5% constituted the *de novo* TE library. Finally, a customized library (combination of homolog and *de novo* TE library without redundancy) was subjected to homology search using RepeatMasker to identify TEs. As a result, extensive repeat sequences including tandem repeats and interspersed repeats were detected in the genome, accounting for approximately 44.56% (400.62 Mb) of the genome (Table 4), which is close to the repeat rate of 47.82% estimated by the genome survey. The tandem repeat sequences were 57.51 Mb in length, accounting for 6.40% of the genome (Table 4).

**Table 4:** Statistics of the repetitive sequences in female genome of *A. fasciatus*.

Repeat type		Length (bp)	Percentage of genome (%)
Tandem repeats		57,508,533	6.40
Interspersed repeats	DNA	61,193,221	6.81
	LINE	23,535,430	2.62
	SINE	304,503	0.03
	LTR	307,821,430	34.24
	Unknown	17,786,267	1.98
	Sum	391,942,474	43.59
Total	400,615,206	44.56	

### Gene prediction and functional annotation

Three strategies were used to predict gene structures in the female genome: homology searching, *ab initio* prediction, and transcriptome-assisted prediction. For homology searching, the homologous protein sequences of *Danio rerio*, *Ctenopharyngodon idella*, *Megalobrama amblycephala*, *Poropuntius huangchuchieni*, *Puntigrus tetrazona*, *Onychostoma macrolepis*, and *Oryzias latipes* were downloaded from NCBI database (https://ftp.ncbi.nlm.nih.gov/genomes/refseq). Protein sequences were aligned to the genome using TBLASTN (v2.2.26; E-value ≤1e^−5^)^34^, and then the matched proteins were aligned to the homologous genome sequences for accurate spliced alignments with GeneWise (v2.4.1)^35^ which was used to predict gene structure contained in each protein region. For gene predication *ab initio*, AUGUSTUS^36^ (v3.2.3), GeneID^37^ (v1.4), GENSCAN^38^ (v1.0) and GlimmerHMM^39^ (v3.04) and SNAP^40^ (2013-11-29) were used in an automated gene prediction pipeline. For RNA-sequencing-assisted prediction, transcriptome read assemblies were generated with Trinity (v2.1.1) for the genome annotation^41^. To optimize the genome annotation, the RNA-Seq reads from different tissues were aligned to genome sequences using HISAT (v2.0.4) with default parameters to identify exon regions and splice positions^42^. The alignment results were then used as the input for Cufflinks (v2.2.1) with default parameters for genome-based transcript assembly^43^. The non-redundant reference gene set was generated by merging genes predicted by three methods with EvidenceModeler (EVM, v1.1.1) and then further annotated with PASA (Program to Assemble Spliced Alignment)^44^. As a result, we identified 27,392 protein-coding genes in the female reference genome (Table 5, Supplemental Fig. 3a).

**Table 5:** Statistics of gene structure prediction in female genome of *A. fasciatus*.

	Gene set	Number	Average length (bp)				Average exonsper gene
			Transcript	CDS	Exon	Intron	
*De novo*	Augustus	39,035	9,555.76	1,149.54	175.83	1,517.93	6.54
	GlimmerHMM	83,903	9,114.02	624.09	147.61	2,630.08	4.23
	SNAP	63,777	13,744.78	752.24	133.85	2,812.34	5.62
	Geneid	30,882	17,990.46	1,362.23	210.29	3,035.59	6.48
	Genscan	31,144	19,877.33	1,547.92	185.85	2,500.96	8.33
Homolog	Omac	23,317	13,961.01	1,563.70	172.87	1,540.88	9.05
	Olat	20,253	12,699.33	1,537.33	182.18	1,500.55	8.44
	Phua	21,627	12,670.04	1,503.51	177.09	1,490.88	8.49
	Cide	26,050	11,008.54	1,472.97	178.86	1,317.91	8.24
	Ptet	22,329	14,103.75	1,652.90	176.95	1,492.71	9.34
	Mamb	23,444	13,599.47	1,625.22	179.36	1,485.39	9.06
	Drer	22,897	13,113.27	1,597.54	180.95	1,471.01	8.83
RNAseq	PASA	51,202	11,825.05	1,305.37	165.25	1,524.75	7.90
	Transcripts	61,053	25,676.79	3,717.49	316.98	2,046.93	11.73
EVM		35,688	12,191.91	1,283.94	172.32	1,690.90	7.45
Pasa-update*		35,359	12,563.62	1,307.12	173.01	1,717.22	7.56
Final set*		27,392	14,935.96	1,552.99	172.13	1,668.25	9.02

EVM: the results combining from the three strategies, Pasa-update: the result rectified by PASA, “*” means the sequences include the UTR (Untranslated Regions) area.

Gene functions were assigned according to the best match by aligning the protein sequences to the Swiss-Prot^45^ (http://www.uniprot.org/) using BLASTP (E-value ≤ 1e-5). The motifs and domains were annotated using InterProScan70^46^ (v5.31) (https://www.ebi.ac.uk/interpro/). The Gene Ontology (GO) IDs for each gene were assigned according to the corresponding InterPro entry. We predicted the protein function by transferring annotations from the closest BLAST hit (E-value ≤ 1e-5) in the Swiss-Prot database and DIAMOND (v0.8.22)/BLAST hit (E-value < 10-5) in the NR database (ftp://ftp.ncbi.nih.gov/blast/db). We also mapped the gene set to a KEGG pathway and identified the best match for each gene^47^. As a result, 96.1% of the predicted 27,392 protein-coding genes have functional annotations (Supplementary Fig. 3b).

For non-coding RNA (ncRNA) annotation, the tRNAs were predicted using the program tRNAscan-SE^48^. Since rRNAs are highly conserved, the rRNA sequences of *Homo sapiens* were chosen as references, and rRNA sequences were predicted using BLASTN (E-value ≤ 1e-5). Other ncRNAs, including miRNAs and snRNAs were identified by searching against the Rfam database with default parameters using the infernal software^49^. Finally, a total of 35,869 ncRNAs were identified including 2,588 miRNAs, 18,386 tRNAs, 12,709 rRNAs, and 2,186 snRNAs (Supplementary Table 2).

Furthermore, the male genome of *A. fasciatus* was also annotated using the annotation result of the female genome as a reference with the liftoff^50^ software, an accurate gene annotation mapping tool, capable of mapping genes from a reference genome to a target genome.

## Data Records

All the raw sequencing data for genome assembly have been deposited in the NCBI database (https://www.ncbi.nlm.nih.gov/bioproject). Specifically, for the female genome, the Illumina WGS data (SRR26993408^51^-SRR26993409^52^), PacBio WGS data (SRR26993393^53^-SRR26993394^54^), transcriptome data (SRR26993400-SRR269934007^55–62^,SRR26993392^63^) and Hi-C data (SRR26993395-SRR26993399^64–68^) were deposited under the BioProject accession number PRJNA1045882. For the male genome, the PacBio WGS data (SRR27126179^69^) and Hi-C data (SRR27588553^70^) were deposited under the BioProject accession number PRJNA1049304. The final files of the assembled genome of *A. fasciatus* have been deposited at GenBank under the accession number JAXUIB000000000 (female)^71^ and JAZDCR000000000 (male)^72^. Meanwhile, all the data including the male and female genome sequences and annotation files are accessible through the Figshare^73^.

## Technical Validation

**Fig. 3:**
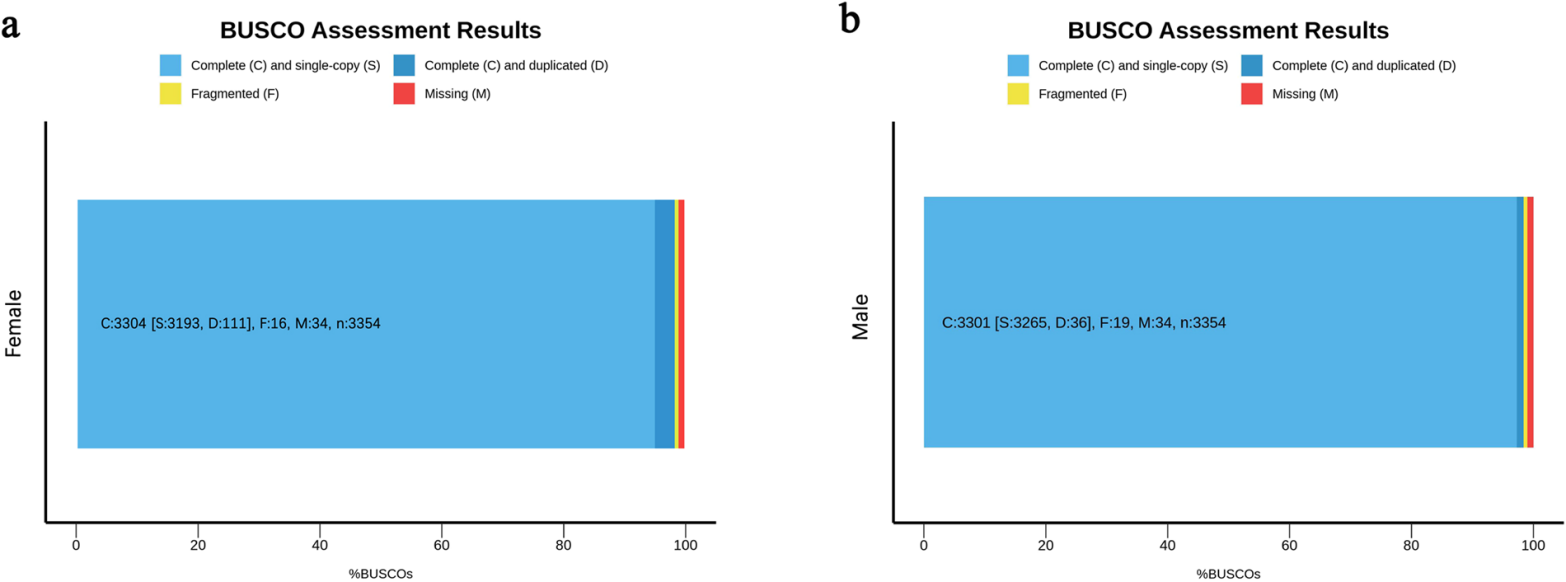
BUSCO assessment results of the genome assembly. (**a**) BUSCO evaluation for the female *A. fasciatus* genome (**b**) BUSCO evaluation for the male *A. fasciatus* genome.

Benchmarking Universal Single-Copy Orthologues (BUSCO)^74^, Core Eukaryotic Genes Mapping Approach (CEGMA)^75^, and Merqury software^76^ were used to evaluate the genome assemblies. The BUSCO (v5.2.2) was used to evaluate the completeness of the genome assemblies with the vertebrata database (vertebrata_odb10). Out of the 3,354 orthologous genes, 3,304 (98.5%) genes were identified as complete genes, 16 (0.5%) genes were identified as fragmented genes, and 34 (1%) genes were missing from the female genome assembly (Fig. 3a). On the other hand, 3,301 (98.5%) genes were identified as complete genes, 19 (0.5%) genes were identified as fragmented genes, and 34 (1%) genes were missing from the male genome assembly (Fig. 3b). Meanwhile, CEGMA (v2.5) evaluation was also considered for genome completeness evaluation. Out of the 248 Eukaryotic core genes, 235 (94.76%) genes and 233 (93.95%) were identified in the female and male genomes, respectively (Supplementary Table 3). To further assess the completeness of genome assemblies, we identified telomeric repeats in both female and male genomes using tidk (v0.2.41) (https://zenodo.org/records/10091385) with Cypriniformes-specific telomeric repeat sequences. The results demonstrated telomeric repeat sequences could be identified in almost all of the chromosome ends (Supplementary Fig. 4). These results indicate an extremely high level of completeness of the genome assemblies.

To evaluate the quality and accuracy of the female genome assembly, we employed a three-step validation process. Firstly, the Illumina short-reads for the genome survey were mapped to genome assembly using BWA-MEM (v0.7.8)^77^ with default parameters, and then SAMtools^77^ was used for SNP calling. As a result, 99.30% of reads were mapped to the genome with approximately 99.95% coverage. Subsequently, the base quality value (QV) of genome sequences was quantified using Merqury software, resulting in a QV score of 52.22. All these results indicate a high-quality genome assembly. The GC skew of genome assembly was calculated with a 10 kb slide window using SOAP.coverage (v2.7.7)^78^. GC content was 37.49% with no obvious separation, indicating no foreign contamination in the genome (Supplementary Fig. 5).

### Supplementary information

Supplementary files

## Data Availability

There were no custom software codes developed. The tools used for reads quality control are non-open scripts developed by the Novogene (Beijing, China). All bioinformatics tools and pipelines were performed following the instructions of the manuals and protocols. The versions of the software used, along with their corresponding parameters, have been thoroughly described in the Methods section.
