# Supplementary material for: Chromosome-scale genome assemblies of sexually dimorphic male and female Acrossocheilus fasciatus
Source: Sci Data. 2024 Jun 21;11:653. doi: 10.1038/s41597-024-03504-9 (PMC11192953; doi:10.1038/s41597-024-03504-9)
Supplement: Supplementary file 1 — Supplementary files [file 41597_2024_3504_MOESM1_ESM.docx]

**Supplementary Figures and Tables**


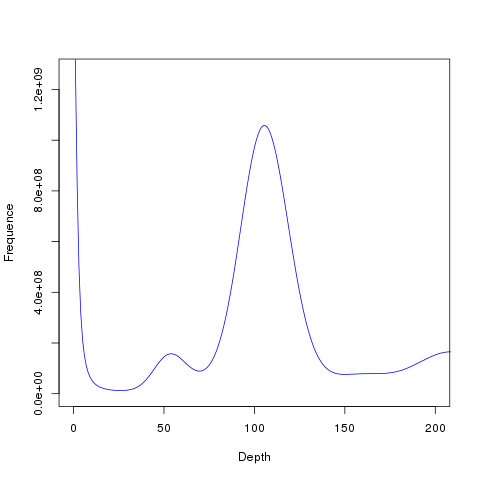


**Supplementary Fig. 1** Frequency map with depth and number of K-mer=17. The horizontal axis is the k-mer depth, and the vertical axis is the number of K-mer corresponding to the depth.


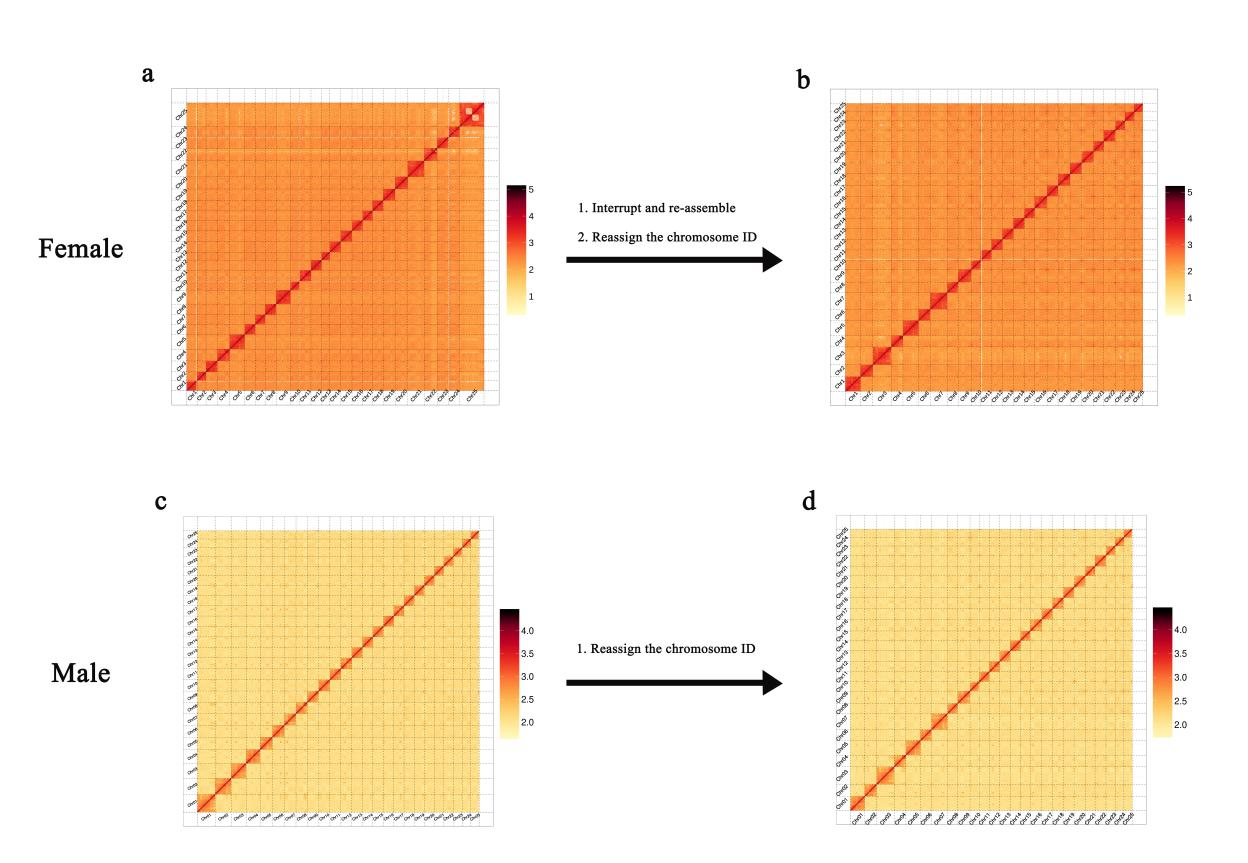


**Supplementary Fig. 2** The Hi-C contact maps before and after manual corrections. Before (a) and after (b) manual corrections of female Hi-C maps. Before (c) and after (d) manual corrections of male Hi-C maps.


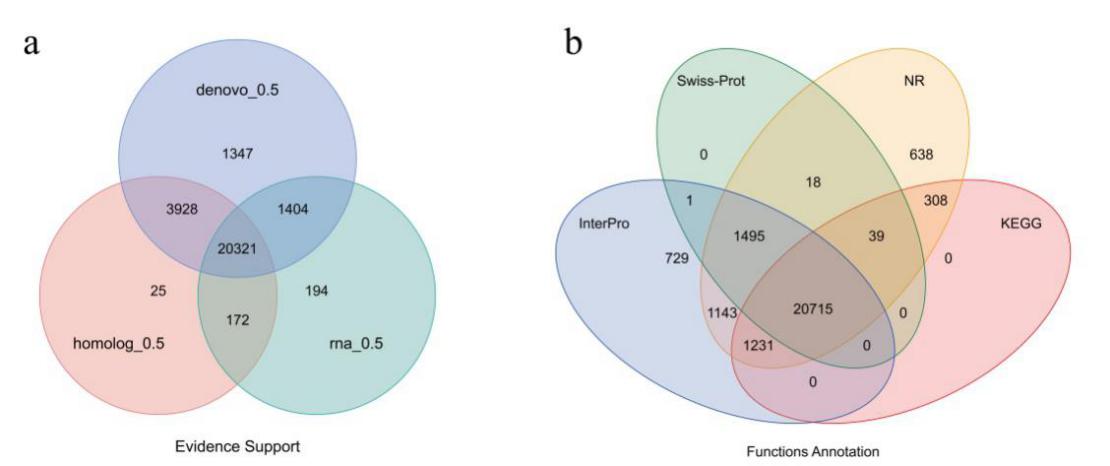


**Supplementary Fig. 3** (a) Venn diagrams of gene set evidence from three prediction methods. The number indicates the number of genes with overlaps greater than 50%. (b)Venn diagram of function annotations from various databases including InterPro, Swiss-Prot, NR and KEGG.


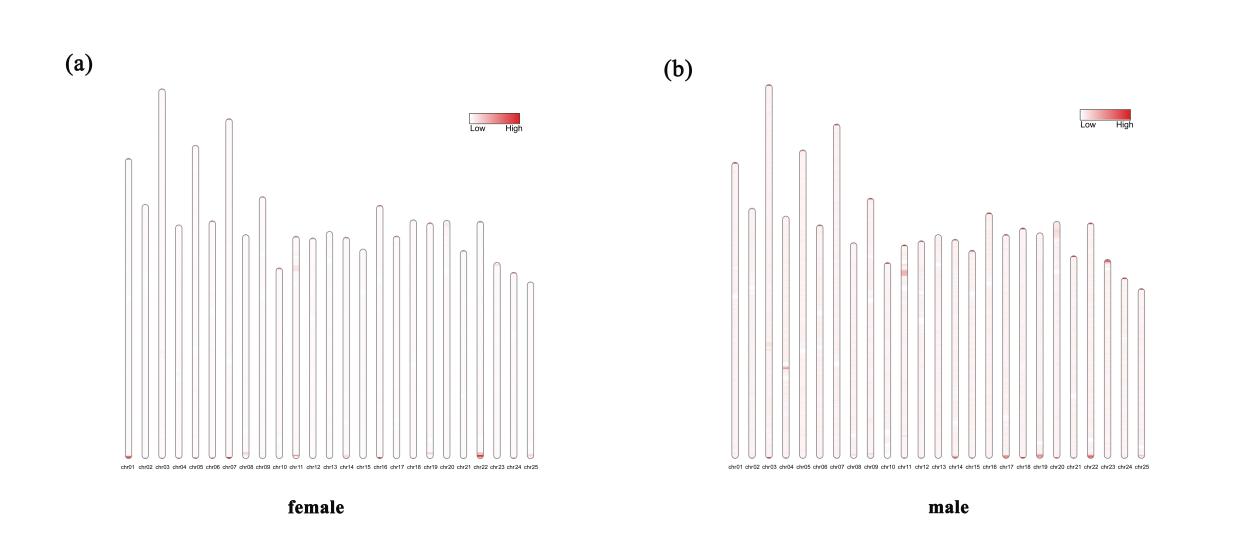


**Supplementary Fig. 4** Distribution maps of telomeric repeat sequences (AACCCT/AGGGTT) on female (a) and male (b) chromosomes.


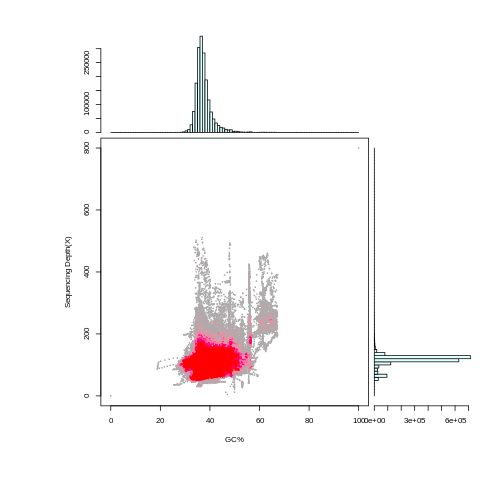


**Supplementary Fig. 5** Distribution map of GC content and depth of the female *A. fasciatus* genome.

**Supplementary Table 1** Statistics of N50 length in female and male genome assemblies of *A. fasciatus*.

|  | Female | Male |
| --- | --- | --- |
| Hifiasm assemblies | 32.58 Mb (contigs) | 33.06 Mb (contigs) |
| Hi-C scaffolding | 32.35 Mb (contigs) | 32.84 Mb (contigs) |
|  | 33.86 Mb (scaffolds) | 33.78 Mb (scaffolds) |

**Supplementary Table 2** Statistics of ncRNAs in the female genome of *A. fasciatus*.

| ncRNA type | | Copy number | Average length (bp) | % of genome |
| --- | --- | --- | --- | --- |
| miRNA | | 2,588 | 119.29 | 0.034336 |
| tRNA | | 18,386 | 75.97 | 0.16 |
| rRNA | rRNA | 12,709 | 128.97 | 0.18 |
|  | 18S | 159 | 729.16 | 0.012894 |
|  | 28S | 484 | 412.60 | 0.022210 |
|  | 5.8S | 60 | 156 | 0.001041 |
|  | 5S | 12,006 | 109.45 | 0.15 |
| snRNA | snRNA | 2,186 | 149.79 | 0.036418 |
|  | CD-box | 325 | 150.33 | 0.005434 |
|  | HACA-box | 88 | 149.82 | 0.001466 |
|  | splicing | 1,722 | 148.93 | 0.028523 |
|  | scaRNA | 44 | 194.57 | 0.000952 |
|  | Unknown | 7 | 55 | 0.000043 |

**Supplementary Table 3** Statistics of CEGMA evaluation

| Genome | Complete | | Complete + Partial | |
| --- | --- | --- | --- | --- |
|  | core gene number | % Completeness | core gene number | %Completeness |
| Female | 232 | 93.55 | 235 | 94.76 |
| Male | 229 | 92.34 | 233 | 93.95 |

(A total of 248 Eukaryotic core genes were built from 6 eukaryons, *Homo sapiens*, *Drosophila melanogaster*, *Arabidopsis thaliana*, *Caenorhabditis elegans*, *Saccharomyces cerevisiae* and *Schizosaccharomyces pombe*).
